# Proline-Modified (RW)*n* Peptides: Enhancing the Antimicrobial Efficacy and Selectivity against Multidrug-Resistant Pathogens

**DOI:** 10.1021/acsomega.4c10757

**Published:** 2025-02-07

**Authors:** Anderson Sunda-Meya, Nsoki Phambu

**Affiliations:** † Department of Physics, Xavier University of Louisiana, New Orleans, Louisiana 70125, United States; ‡ Department of Chemistry, Tennessee State University, Nashville, Tennessee 37209, United States

## Abstract

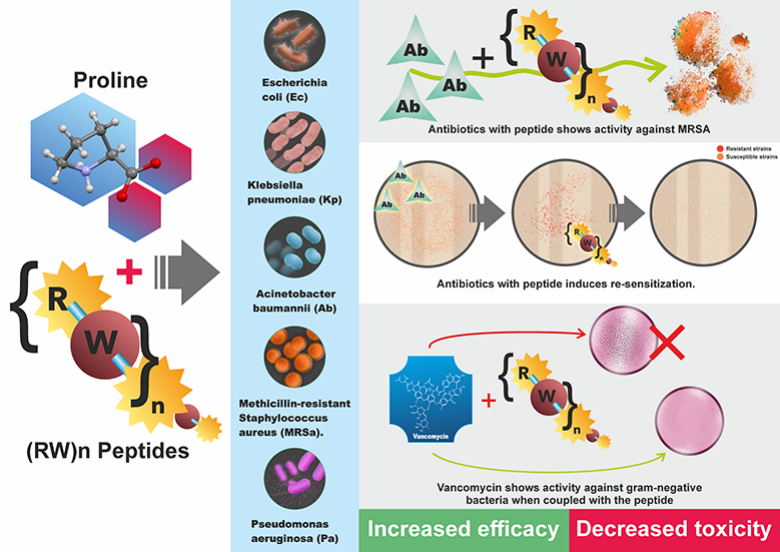

The growing threat of multidrug-resistant (MDR) bacteria necessitates the development of novel antimicrobial agents. This study investigates the potential of proline-modified (RW)*n* peptides as a platform for combating MDR pathogens with minimal toxicity. We synthesized and evaluated (RW)*n* peptides (*n* = 4, 6, and 8) with and without central proline residues against five clinically relevant bacterial strains, including ESKAPE pathogens. Antimicrobial activity, cytotoxicity, and synergistic effects with conventional antibiotics were assessed. Proline incorporation significantly enhanced the peptide selectivity and broadened the spectrum of activity, particularly against Gram-negative bacteria. RW6P and RW8P demonstrated exceptional efficacy (MICs ≤ 0.25 μg/mL) against methicillin-resistant *Staphylococcus aureus* and *Escherichia coli* with minimal toxicity to human cells. Notably, RW8P restored ampicillin susceptibility in *Pseudomonas aeruginosa* (MIC < 0.25 μg/mL) without dose-dependent toxicity. Exceptionally, RW6-2P demonstrated efficacy against all Gram-positive and Gram-negative bacteria, except *Klebsiella pneumoniae* (Kp), without toxicity. Furthermore, several Gram-negative isolates were rendered susceptible to vancomycin when combined with these peptides, addressing a key limitation of glycopeptide antibiotics. Gram-negative *Klebsiella pneumoniae* (Kp) was rendered susceptible to vancomycin when combined with RW4P and RW6-2P. RW4P and RW6-2P, with and without antibiotics, have shown selectivity. This study presents proline-modified (RW)*n* peptides as promising candidates for developing broad-spectrum antimicrobials with enhanced selectivity and the potential to revitalize existing antibiotics against MDR pathogens.

## Introduction

Antimicrobial resistance (AMR) represents one of the most pressing global health challenges of the 21st century.^1,2^ The rapid emergence and spread of multidrug-resistant (MDR) bacteria threaten to undermine decades of progress in infectious disease treatment, potentially ushering in a postantibiotic era where common infections once again become life-threatening.^1−7^ The World Health Organization has declared AMR a top-ten global public health threat, with projections indicating that it could cause 10 million deaths annually by 2050 if left unchecked.^2,7^ The gravity of this situation is underscored by recent data: in 2019, AMR was associated with an estimated 4.95 million deaths globally, with approximately 1.27 million deaths directly attributed to bacterial AMR.^1,2^ This crisis spans both developed and developing nations, highlighting the urgent need for innovative global solutions. Despite the approval of new antimicrobial drugs each year, many are merely modifications of existing compounds and prove ineffective against extensively drug-resistant (XDR) or pan-drug-resistant (PDR) bacterial strains.^1,8^

In response to this growing threat, the scientific community has mobilized to explore novel approaches to combat AMR. Antimicrobial peptides (AMPs) have emerged as a promising avenue of research due to their potent antibacterial activities and unique mechanisms of action. As part of the innate immune system of various organisms, AMPs have evolved to combat a wide range of pathogens, including many MDR strains.^1,2,4,6,9−14^ However, their clinical application faces challenges related to stability, bioavailability, toxicity, and production costs.^5,6,9,14^

Our research focuses on a specific class of AMPs: the (RW)*n* peptide series. These short peptides, composed of alternating arginine (R) and tryptophan (W) residues, have demonstrated remarkable antimicrobial efficacy even at very short lengths.^8,15^ Building on this foundation, we propose a novel approach: the incorporation of proline residues into the (RW)*n* series to enhance their selectivity and broaden their spectrum of activity while minimizing their toxicity.

This study aims to address several critical gaps in current AMP research: (i) we investigate the antibacterial effectiveness of *N*-terminal (RW)*n* peptides against five clinically relevant bacterial strains, including ESKAPE pathogens – *Enterococcus faecium*, *Staphylococcus aureus* (MRSA), *Klebsiella pneumoniae* (Kp), *Acinetobacter baumannii* (Ab), *Pseudomonas aeruginosa* (Pa), and Enterobacter species – known for their multidrug resistance. (ii) We explore how proline modification of the (RW)*n* series can enhance peptide safety and broaden antibacterial activities, potentially overcoming the limitations of current AMPs. (iii) We assess the potential of (RW)*n* peptides as adjuvants to conventional antibiotics,^16^ specifically examining their ability to resensitize resistant bacteria to penicillin (PEN), vancomycin (VAN), and ampicillin (AMP).

Our methodological approach combines peptide synthesis, antimicrobial susceptibility testing, and toxicity assays. We utilize the Community for Open Antimicrobial Drug Discovery (CO-ADD)^17^ platform to evaluate our peptides against a panel of bacterial strains, ensuring standardized and comparable results.

**Figure 1:**
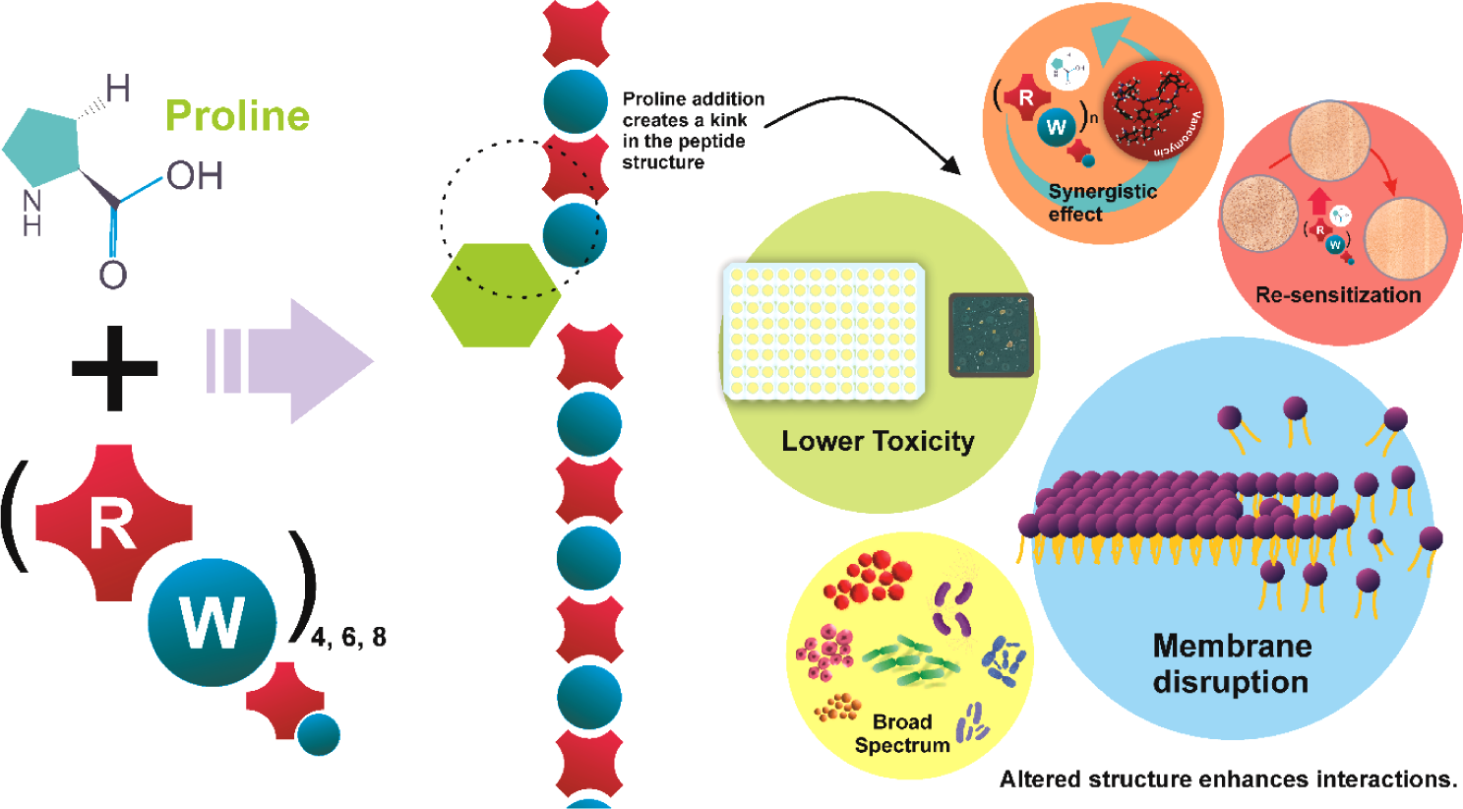
Schematic representation of (RW)*n* peptides and the effect of proline modification on their structure.

We hypothesize (Figure 1) that proline-modified (RW)*n* peptides will demonstrate enhanced selectivity for bacterial membranes, broader spectrum activity, and reduced toxicity compared to their unmodified counterparts. Furthermore, we anticipate that these peptides will synergize with conventional antibiotics, potentially revitalizing the efficacy of existing drugs against resistant strains.

Focusing on these objectives, we aim to contribute to the development of novel antimicrobial strategies that can effectively combat the growing threat of antibiotic-resistant bacteria. Our approach offers a promising path forward in the urgent quest for new weapons against AMR, potentially paving the way for a new class of antimicrobial agents that combine broad-spectrum activity with minimal toxicity.

This research not only addresses the immediate need for new antimicrobial compounds but also contributes to our understanding of peptide-based therapeutics, potentially opening new avenues for drug design in the ongoing battle against antimicrobial resistance.

## Experimental Methods

### Peptide Synthesis and Characterization

The C-amidated (RW)*n* peptides (*n* = 4, 6, 8) and their proline-modified variants (RW4P, RW6P, RW8P, RW6-2P) were synthesized by GenScript Inc., New Jersey, USA, using standard solid-phase peptide synthesis techniques.^18^ Peptides were purified by reverse-phase high-performance liquid chromatography (HPLC) and characterized by mass spectrometry. Purity (>95%) was confirmed by analytical HPLC.

### Antimicrobial Activity Assays

Antimicrobial activity was evaluated using the Community for Open Antimicrobial Drug Discovery (CO-ADD) screening platform. Minimum inhibitory concentrations (MICs) were determined against the following five bacterial strains: *Escherichia coli* (*E. coli*) ATCC 25922, *Klebsiella pneumoniae* ATCC 700603, *Acinetobacter baumannii* ATCC 19606, *Pseudomonas aeruginosa* ATCC 27853, and *Staphylococcus aureus* ATCC 43300.

### Sample Preparation

Peptides were prepared in dimethyl sulfoxide (DMSO) and water to a final testing concentration of 32 μg/mL or 20 μM in 384-well, nonbinding surface plates (NBS) for each bacterial strain, in duplicate (*n* = 2), with a maximum final DMSO concentration of 1%.

### Bacterial Assay

Bacteria were cultured in cation-adjusted Mueller–Hinton broth (CAMHB) at 37 °C overnight. Cultures were diluted 40-fold in fresh broth and incubated at 37 °C for 1.5–3 h. Mid-log phase cultures were then diluted and added to the compound-containing plates, giving a final cell density of 5 × 10^5^ CFU/mL in a total volume of 50 μL. Plates were incubated at 37 °C for 18 h without shaking.

### Analysis

Bacterial growth inhibition was determined by measuring the absorbance at 600 nm (OD600) using a Tecan M1000 Pro monochromator plate reader. The percentage of growth inhibition was calculated using negative (media only) and positive (bacteria without inhibitors) controls. Significance was determined by modified *Z* scores calculated using the median and MAD of the samples on the same plate. Samples with inhibition values above 80% and *Z* scores above 2.5 for either replicate were classified as active.

### Cytotoxicity Assays

Cytotoxicity was evaluated using human embryonic kidney (HEK293) and human red blood cells (RBCs). HEK293 cells were cultured in DMEM supplemented with 10% FBS. Cells were seeded in 384-well plates and incubated with peptides for 24 h. Cell viability was assessed using the resazurin reduction assay. Hemolysis assays were performed using fresh human RBCs incubated with peptides for 1 h at 37 °C.

### Synergy Studies

Synergistic effects were evaluated by combining (RW)*n* peptides with conventional antibiotics (PEN, VAN, and AMP) against selected bacterial strains. The fractional inhibitory concentration (FIC) index was calculated to determine synergy, additivity, or antagonism.

### Quality Control and Data Analysis

Colistin and VAN were used as positive bacterial inhibitor standards for Gram-negative and Gram-positive bacteria, respectively. The quality control (QC) of the assays was determined by the antimicrobial controls and the *Z*’ factor (using positive and negative controls). Plates with a *Z*’ factor of above 0.4 and antimicrobial standards showing the expected range of activity were considered to pass QC.

Percentage growth inhibition was calculated using negative (media only) and positive (bacteria without inhibitors) controls. Modified *Z* scores were used to determine the significance of the inhibition values. Samples with inhibition values of above 80% and *Z* scores of above 2.5 for either replicate were classified as active. Data are presented as the mean ± standard deviation from at least three independent experiments.

## Results and Discussion

This study investigates the antimicrobial efficacy and adjuvant potential of proline-enhanced (RW)*n* peptides against MDR bacteria. We address three key questions: (1) what is the antibacterial efficacy of *N*-terminal (RW)*n* peptides against five clinically relevant bacterial strains, (2) how does proline modification influence peptide safety and broaden the spectrum of antibacterial activity, and (3) can these peptides synergize with conventional antibiotics to resensitize resistant strains? Our findings demonstrate the promising capabilities of these peptides in combating antimicrobial resistance and provide insights into their mechanisms of action. We will discuss our results in the context of the current literature on AMPs and explore their implications for future antimicrobial strategies. The following sections present a comprehensive analysis of the peptides’ structural properties, antimicrobial activity, toxicity profiles, and synergistic effects with conventional antibiotics.

### Structural and Physicochemical Properties of (RW)*n* Peptides

The design of the (RW)*n* peptides was driven by the need to optimize their amphipathic properties, which are essential for effective antimicrobial action. Amphipathic peptides, characterized by distinct hydrophilic and hydrophobic regions, are known to interact with bacterial membranes through electrostatic forces and hydrophobic interactions, leading to membrane disruption. The alternating arginine (R) and tryptophan (W) residues in the (RW)*n* series are crucial for generating these amphipathic properties. Arginine contributes positively charged guanidinium groups, which are known to interact with the negatively charged components of bacterial membranes, while tryptophan provides hydrophobicity and is associated with membrane insertion due to its affinity for lipid bilayers.^19^ These design choices aim to create peptides that can efficiently target and disrupt bacterial membranes without significantly harming mammalian cells.

Table 1 presents the key structural and physicochemical properties of the (RW)*n* peptides, including the peptide length, net charge, hydrophobicity, and amphipathic index. As the peptide length increases from RW4 to RW8, so does the overall hydrophobic surface area and net positive charge. This balance of charge and hydrophobicity is crucial for determining the peptides’ ability to interact with and disrupt bacterial membranes and also plays a role in their potential cytotoxicity to mammalian cells.

**Table 1:** Key Structural and Physicochemical Properties of (RW)*n* Peptides

Peptide	Length (*n*)	Net charge	Hydrophobicity index	Amphipathic index
RW4	4	+4	Moderate	High
RW6	6	+6	High	Higher
RW8	8	+8	Very High	Highest

The increasing net positive charge with peptide length – from +4 for RW4 to +8 for RW8 – plays a critical role in the peptides’ antimicrobial efficacy. The higher positive charge enhances electrostatic interactions between the peptide and the negatively charged bacterial membrane components, such as lipopolysaccharides in Gram-negative bacteria and teichoic acids in Gram-positive bacteria. These interactions are pivotal in enabling the peptides to bind to the bacterial surface and initiate membrane disruption, ultimately leading to bacterial cell death. As a result, longer peptides, such as RW6 and RW8, are expected to display increased antimicrobial activity compared to shorter peptides like RW4.^12,20,21^

However, the relationship between the charge and antimicrobial activity is not straightforward. While higher net charges can increase the likelihood of bacterial membrane disruption, they may also raise concerns regarding toxicity to mammalian cells. Mammalian cell membranes, although less negatively charged than bacterial membranes, still contain anionic phospholipids and cholesterol, which could interact with highly cationic peptides. Therefore, as the charge of the peptide increases, so does the risk of nonspecific interactions with mammalian cells, which can lead to cytotoxic effects.^22,23^ This necessitates a careful balance between maximizing antimicrobial efficacy and maintaining selectivity to minimize harm to host cells.

The relationship between the peptide length and amphipathicity also affects the membrane-disruptive potential of the peptides. As the length of the peptide increases, its amphipathic nature becomes more pronounced, which enhances its ability to insert into lipid bilayers. This makes longer peptides like RW8 more effective at causing membrane destabilization and lysis. However, the increase in hydrophobicity and amphipathicity with peptide length could also contribute to higher cytotoxicity, as highly amphipathic peptides may insert into and disrupt not only bacterial membranes but also mammalian cell membranes.^24,25^

Thus, while longer peptides in the (RW)*n* series are expected to be more potent against bacteria due to their enhanced electrostatic and hydrophobic interactions, they may also require more careful consideration of dosage and structural modifications to ensure that their toxicity remains within acceptable limits for therapeutic applications. This delicate balance highlights the importance of optimizing peptide properties for maximum efficacy and minimal toxicity.^26^

By systematically exploring the interplay between peptide length, charge, and amphipathicity, this study aims to identify peptide designs that offer robust antimicrobial activity while maintaining selectivity for bacterial cells over mammalian cells.

### Gram-Positive Activity

All (RW)*n* peptides demonstrated potent antimicrobial activity against MRSA, with RW6 exhibiting the lowest minimum inhibitory concentration (MIC) at ≤0.25 μg/mL. This remarkable efficacy against Gram-positive bacteria aligns with prior studies of cationic AMPs, which are characterized by their amphipathic nature and positive charge. These properties enable them to interact strongly with the negatively charged bacterial membranes, resulting in rapid membrane disruption and bacterial cell death. The superior performance of RW6 underscores the importance of optimizing peptide length, charge, and hydrophobicity to achieve maximum efficacy. These factors play a critical role in membrane interaction, with RW6 striking an ideal balance that makes it particularly effective against Gram-positive pathogens, including MRSA, a major cause of hospital-acquired infections.

The MIC data for the (RW)*n* peptides against MRSA (Table 2) highlight a trend in antimicrobial activity, with RW6 and all proline-modified peptides showing the highest efficacy. This trend is consistent with findings from previous studies by Strøm et al., who indicated that the optimal length for antimicrobial peptides generally falls within the range of 12 to 18 amino acids.^27^ Peptides in this range achieve an optimal balance between charge density and hydrophobicity, which is critical for effective interaction with bacterial membranes. RW6, with a length of 12 amino acids, aligns well with this optimal range, explaining its superior efficacy compared with RW4 and RW8.

**Table 2:** Minimum Inhibitory Concentrations (MICs) of (RW)*n* Peptides against MRSA^a^

Peptide	MIC (μg/mL)
RW4	8
RW6	≤0.25
RW8	2
RW4P	≤0.25
RW6P	≤0.25
RW8P	≤0.25
RW6–2P	≤0.25

a All values are MICs in μg/mL.

Interestingly, the introduction of proline residues into the (RW)*n* peptide sequence, as seen in RW4P, RW6P, RW8P, and RW6-2P, uniformly improved antimicrobial activity against MRSA, achieving MICs of ≤0.25 μg/mL across all lengths without toxicity. The enhancement of activity in proline-modified peptides can be attributed to the structural kinks introduced by proline, which likely promotes better membrane penetration and disruption. Proline-induced bends in the peptide structure disrupt the regular α-helical or β-sheet conformation, creating a more flexible structure that may enhance the interaction with bacterial membranes.^28,29^ This structural modification appears to mitigate the length-dependent variations in activity observed in the nonmodified peptides, suggesting that proline incorporation could be a valuable strategy for optimizing antimicrobial peptides for Gram-positive bacterial infections.^30,31^

The enhanced activity of the proline-modified peptides is particularly noteworthy, given the ongoing global health challenge posed by antibiotic-resistant bacteria such as MRSA. With antibiotic resistance on the rise, there is an urgent need for new therapeutic strategies that can combat these resistant strains. The low MIC values achieved by RW6 and its proline-modified variants suggest that these peptides could serve as effective antimicrobial agents in the fight against resistant Gram-positive bacteria. Their ability to achieve such low MICs, even at relatively short peptide lengths, highlights their therapeutic potential, particularly in environments where traditional antibiotics are becoming less effective.

The findings presented here underscore the importance of fine-tuning the structural and physicochemical properties of antimicrobial peptides to maximize their efficacy. The high efficacy of RW6 and the proline-modified peptides against MRSA suggests that these peptides have significant potential as novel therapeutic agents, especially against resistant strains. However, further research is needed to explore their full therapeutic potential, particularly in the context of in vivo models, where factors such as stability, bioavailability, and immunogenicity must be thoroughly evaluated.

### Gram-Negative Activity

RW6 demonstrated broad-spectrum activity, effectively inhibiting *E. coli* with an MIC of ≤0.25 μg/mL. This suggests that the peptide’s structural properties enable it to overcome some of the barriers presented by the Gram-negative outer membrane, consistent with findings by Shai et al.^32^ on the importance of amphipathicity in membrane penetration. The longer RW8 peptide inhibited both *E. coli* and Ab with an MIC of 32 μg/mL. The reduced activity of RW8 compared to RW6 against *E. coli* indicates that the increasing peptide length may impede its ability to penetrate the outer membrane of some Gram-negative bacteria, possibly due to steric hindrance or reduced flexibility in crossing the outer lipopolysaccharide layer. This aligns with observations by Tossi et al.^33^ on the relationship between peptide length and antimicrobial activity. Conversely, the increased activity of RW8 relative to RW6 against Ab suggests that longer peptides may be more effective against certain Gram-negative strains, as noted by Hancock and Sahl^34^ in their review of antimicrobial peptides. These contrasting results highlight that the peptide length, structure, and bacterial strain type are critical determinants of efficacy against Gram-negative pathogens. An optimal balance must be struck to achieve effective membrane penetration while maintaining antimicrobial potency, as emphasized by Brogden^35^ in his work on antimicrobial peptide mechanisms.

The results in Table 3 reveal a complex relationship between peptide structure and activity against Gram-negative bacteria. RW6 demonstrates exceptional activity against *E. coli*, with an MIC of ≤0.25 μg/mL, significantly outperforming both shorter (RW4) and longer (RW8) peptides. This aligns with findings by Tossi et al.,^33^ who reported that the optimal length for antimicrobial peptides often falls between 12 and 18 amino acids.

**Table 3:** MICs of (RW)*n* Peptides against Gram-Negative Bacteria^a^

Peptide	*E. coli*	Kp	Pa	Ab
RW4	32	>32	>32	>32
RW6	≤0.25	>32	>32	>32
RW8	32	>32	>32	32
RW4P	≤0.25	>32	>32	>32
RW6P	≤0.25	>32	>32	>32
RW8P	16	>32	>32	8

a All values are MICs in μg/mL.

Interestingly, the introduction of proline residues (RW4P, RW6P, RW8P) appears to enhance activity against *E. coli* and Ab. This improvement is particularly notable for RW4P and RW8P, suggesting that proline-induced structural changes may facilitate better outer membrane penetration. These findings are consistent with the work by Shai and Oren,^36^ who demonstrated that proline residues can enhance the selectivity and potency of antimicrobial peptides against Gram-negative bacteria.

However, all peptides, except RW6-2P, showed limited efficacy against Kp and Pa, with MICs > 32 μg/mL. This resistance aligns with observations by Hancock and Brinkman,^37^ who noted that these species possess highly effective efflux pumps and modified outer membrane structures that contribute to their intrinsic resistance to many antimicrobial agents.

The improved activity of proline-modified peptides, particularly against Ab, supports the hypothesis that structural flexibility plays a crucial role in overcoming the outer membrane barrier of Gram-negative bacteria. This is in line with research by Brogden,^35^ who emphasized the importance of conformational flexibility in antimicrobial peptide activity.

These results highlight the complex interplay among peptide length, structure, and antimicrobial activity against Gram-negative bacteria. While RW6 shows promise against *E. coli*, the broader challenge of developing peptides effective against a wide range of Gram-negative pathogens remains. The potential of proline modifications to enhance activity, particularly against resistant strains such as Ab, warrants further investigation and may provide a valuable strategy for developing more effective antimicrobial peptides.

### Toxicity and Therapeutic Potential

A key finding of this study is the favorable toxicity profile of the (RW)*n* peptides, particularly, RW6 and RW8. Both peptides demonstrated low toxicity to human HEK293 cells, with MIC values exceeding 32 μg/mL. The low toxicity of RW6 and RW8 suggests that these peptides hold substantial therapeutic potential, especially given their potent antimicrobial activity against both Gram-positive and Gram-negative bacteria. The selective targeting of bacterial membranes while sparing mammalian cells makes these peptides promising candidates for further preclinical evaluation.

The results in Table 4 demonstrate the good safety profile of the (RW)*n* peptides. Notably, all (RW)*n* peptides show minimal toxicity to HEK293 cells (MIC > 32 μg/mL), indicating a high degree of selectivity for bacterial cells over mammalian cells. This selectivity is crucial for the development of antimicrobial agents with minimal side effects, as highlighted by Zasloff in his review of antimicrobial peptides as therapeutic agents.^38^

**Table 4:** Toxicity Profile of (RW)*n* Peptides and Indolicidin^a^

Peptide	HEK293 cells MIC (μg/mL)	RBC hemolysis MIC (μg/mL)
RW4	>32	>32
RW6	>32	≤0.25
RW8	>32	0.4523
RW4P	>32	>32
RW6P	>32	>32
RW8P	>32	>32
RW6–2P	>32	>32
Indolicidin	>32	>32

a All values are MICs in μg/mL.

The hemolytic activity of the peptides varies, with RW6 and RW8 showing some hemolytic potential (MIC ≤ 0.25 μg/mL and 0.4523 μg/mL, respectively), while RW4 and all proline-modified peptides (RW4P, RW6P, RW8P) exhibit no significant hemolytic activity (MIC > 32 μg/mL). In addition, RW6-2P, alone or in combination, exhibits no hemolicity and no cytotoxicity. This observation aligns with findings by Dathe et al., who reported that the incorporation of proline residues in antimicrobial peptides can reduce hemolytic activity while maintaining antimicrobial efficacy.^39^ However, proline incorporation in antimicrobial peptides can also disrupt the alignment of amino acid side chains, potentially altering their amphipathicity and impacting their membrane-targeting efficiency.^40^

The low toxicity of proline-modified peptides is particularly noteworthy. As discussed by Shai and Oren, proline residues can induce kinks in peptide structures, potentially enhancing their selectivity for bacterial membranes over mammalian cell membranes.^36^ This structural modification appears to have successfully mitigated the hemolytic activity observed in RW6 and RW8 while preserving their antimicrobial properties.

In comparison, Shai et al. in their studies on indolicidin analogs^36^ noted that indolicidin showed a higher toxicity profile that has been a limiting factor in the clinical development of indolicidin.

The therapeutic potential of the (RW)*n* peptides, especially the proline-modified variants, is further underscored by their broad-spectrum activity against both Gram-positive and Gram-negative bacteria, as shown in previous sections. This combination of potent antimicrobial activity and low toxicity addresses a critical need in the field of antimicrobial peptide development, as emphasized by Hancock and Sahl in their review of antimicrobial peptides as new anti-infective therapeutic strategies.^34^

Moreover, the ability of these peptides to maintain their antimicrobial efficacy while exhibiting low toxicity suggests that they may have a therapeutic window wider than that of conventional antibiotics. This characteristic is particularly valuable in the context of treating systemic infections, where higher doses may be required to achieve therapeutic effects.

Future studies should focus on in vivo toxicity assessments and the pharmacokinetic profiling of these peptides to further evaluate their potential for clinical development. Additionally, investigating the mechanism by which the proline-modified peptides maintain their selectivity for bacterial cells could provide valuable insights into the design of next-generation antimicrobial peptides with enhanced safety profiles.

### Structure–Activity Relationships

Our results confirm previous findings on the stability and antimicrobial activity of the (RW)*n* peptides.^20^ The trend in antimicrobial activity (RW6 > RW8 > RW4) correlates with earlier biophysical studies, which indicated that an optimal peptide length exists for balancing stability, antimicrobial efficacy, and toxicity. Specifically, as shown in Table 5, RW6 appears to offer the best combination of structural stability and membrane-disruptive properties, while RW4’s shorter length and lower hydrophobicity limit its effectiveness. Conversely, RW8, though more potent than RW4, shows signs of decreased efficacy against some types of Gram-negative bacteria, suggesting that its longer chain may hinder effective membrane interaction in certain cases.

**Table 5:** Structure–Activity Relationships of (RW)*n* Peptides^a^

Peptide	Length	Antimicrobial activity	Toxicity	Thermal stability
RW4	Short	Moderate	Low	Low
RW6	Medium	High	Moderate	High
RW8	Long	High (Gram+), Moderate (Gram−)	Moderate	High

a All values are MICs in μg/mL.

The structure–activity relationships observed in our study align with the findings of Chen et al., who reported that the optimal length for antimicrobial peptides often falls between 12 and 18 amino acids.^41^ This range allows for sufficient amphipathicity and membrane-disruptive potential while minimizing self-aggregation and toxicity issues associated with longer peptides.

RW6’s superior performance can be attributed to its optimal balance of charge density and hydrophobicity. As noted by Dathe and Wieprecht, these factors are crucial in determining the selectivity and potency of antimicrobial peptides. The alternating arginine and tryptophan residues in RW6 create an ideal amphipathic structure that can effectively interact with and disrupt bacterial membranes.^42^

The reduced efficacy of RW8 against some Gram-negative bacteria, despite its higher overall potency, is consistent with observations by Tossi et al.^33^ They found that longer peptides may face challenges in penetrating the outer membrane of Gram-negative bacteria due to steric hindrance or reduced flexibility. This phenomenon highlights the importance of considering not just the overall antimicrobial activity but also the spectrum of activity when designing antimicrobial peptides. The lower activity of RW4 can be explained by its shorter length, which may limit its ability to form stable membrane-spanning structures. This aligns with the work of Shai and Oren, who demonstrated that a minimum peptide length is often necessary to achieve optimal membrane permeabilization and antimicrobial activity.^36^

These structure–activity relationships provide valuable insights for the rational design of antimicrobial peptides. They suggest that future peptide engineering efforts should focus on optimizing the balance between charge, hydrophobicity, and length to achieve broad-spectrum activity while minimizing toxicity.

### Proline Modification Effects

The incorporation of proline into the (RW)*n* sequences (RW4P, RW6P, RW8P, and RW6-2P) significantly altered their antimicrobial profiles. Proline residues introduce structural kinks in the peptide backbone, which may enhance the selectivity for bacterial membranes over mammalian membranes. This structural modification likely disrupts the uniformity of the peptide’s amphipathic structure, resulting in greater flexibility and enabling more efficient membrane interaction with bacterial cells. Because RW6 appears to offer the best combination of structural stability and membrane-disruptive properties in our previous studies,^20^ we incorporated two proline residues in RW6 to generate RW6-2P with the sequence RWRW-P-RWRW-P-RWRW.

The results in Table 6 demonstrate the significant impact of proline modification on the antimicrobial activity and toxicity profiles of the (RW)*n* peptides. Notably, RW8P exhibited improved activity against Ab (MIC = 8 μg/mL), a Gram-negative pathogen. This enhanced activity suggests that proline-induced kinks may facilitate better penetration of Gram-negative membranes, particularly in pathogens with more rigid outer membranes.

**Table 6:** Antimicrobial Activity and Toxicity of the Proline-Modified (RW)*n* Peptides^a^

Peptide	MRSA	*E. coli*	Kp	Pa	Ab	HEK293	RBC
RW4P	≤0.25	≤0.25	>32	>32	>32	>32	>32
RW6P	≤0.25	≤0.25	>32	>32	>32	>32	>32
RW8P	≤0.25	>32	>32	>32	8	>32	>32
RW6-2P	≤0.25	16	>32	≤0.25	2	>32	>32

a All values are MICs in μg/mL.

The introduction of proline residues has been shown to enhance the selectivity of antimicrobial peptides for bacterial membranes in mammalian cells. This effect is attributed to the unique structural properties of proline, as reported by Shai and Oren.^36^ They found that proline-containing peptides maintain conformational flexibility, which improves their potency against bacteria while reducing their hemolytic activity.

Furthermore, the positioning of proline residues is crucial for maintaining optimal antibacterial activity and resisting structural ordering, as demonstrated by Vermeer et al.^43^ Their study showed that proline residues can disrupt the α-helical structure of antimicrobial peptides, leading to enhanced membrane permeabilization and selectivity.

The improved activity of RW8P against Ab is particularly noteworthy as this pathogen is known for its intrinsic resistance to many antibiotics. This finding aligns with research by Kang et al., who reported that proline-rich antimicrobial peptides can target intracellular pathways in addition to membrane disruption, making them promising candidates for next-generation antibiotics.^44^

The ability of proline-modified peptides to maintain or even enhance antimicrobial efficacy while reducing cytotoxicity makes them strong candidates for further investigation as therapeutic agents. This is supported by the work of Brogden, who emphasized the importance of peptide flexibility in antimicrobial activity and selectivity.^35^

In summary, the proline modification of (RW)*n* peptides appears to offer a promising strategy for developing antimicrobial peptides with enhanced selectivity and broader spectrum activity, particularly against challenging Gram-negative pathogens. Future studies should focus on optimizing the position and the number of proline residues to further improve antimicrobial efficacy while maintaining low toxicity to mammalian cells.

### Synergistic Effects with Conventional Antibiotics

Combining (RW)*n* peptides with conventional antibiotics revealed promising synergistic effects. The results in Table 7 demonstrate the significant synergistic effects observed when (RW)*n* peptides are combined with conventional antibiotics. This synergy is particularly evident with the proline-modified RW6P. All of the results are available in the Supporting Information.

**Table 7:** Synergistic Effects of (RW)*n* Peptides (with and without Proline) with Conventional Antibiotics without Toxicity (Component in Capital Letter Means 95 mg and Component in Minor Letter Means 5 mg)^a^

Combination	MRSA	*E. coli*	Kp	Pa	Ab
PEN	8	32	>32	>32	>32
AMP	32	8	>32	>32	>32
VAN	≤0.25	>32	>32	>32	>32
PEN-rw4p	2	32	>32	>32	>32
AMP-rw4p	2	8	>32	>32	>32
VAN-rw4p	≤0.25	32	32	>32	16
PEN-rw6p	≤0.25	>32	>32	>32	>32
AMP-rw6p	8	32	>32	>32	>32
VAN-rw6p	≤0.25	>32	>32	>32	>32
AMP-rw8p	2	8	>32	≤0.25	>32
RW6P-pen	≤0.25	>32	>32	>32	>32
RW6P-amp	≤0.25	≤0.25	>32	>32	>32
RW6P-van	≤0.25	≤0.25	>32	>32	8

a All values are MICs in μg/mL.

The combination of RW6P with VAN (VAN-rw6p) showed exceptional activity against both Gram-positive (MRSA) and Gram-negative (*E. coli*) bacteria, with MICs ≤ 0.25 μg/mL. This is a notable improvement, especially for *E. coli*, as VAN alone is typically ineffective against Gram-negative bacteria due to its inability to penetrate the outer membrane. This synergistic effect aligns with findings by Briers et al., who reported that membrane-active peptides can enhance the efficacy of antibiotics against Gram-negative pathogens.^45^

Similarly, the combination of RW8P with AMP (AMP-rw8p) demonstrated potent activity against MRSA, *E. coli*, and notably Pa (MIC ≤ 0.25 μg/mL). The efficacy against Pa is particularly significant, as this pathogen is known for its intrinsic resistance to many antibiotics. This result is consistent with research by Zhanel et al., who found that combining AMPs with β-lactam antibiotics can overcome resistance mechanisms in Pseudomonas species.^46^ Also, the combination of RW4P with VAN (VAN-rw4p) demonstrated potent activity against MRSA, *E. coli*, Kp, and Ab.

The observed synergistic effects suggest that these combinations could revitalize the efficacy of existing antibiotics against resistant strains, potentially offering a novel strategy to counteract the growing issue of antimicrobial resistance. This approach aligns with the concept of antibiotic adjuvants, as described by Wright, where nonantibiotic compounds are used to enhance the efficacy of existing antibiotics.^47^

The use of such combinations could also allow for lower doses of antibiotics, reducing the risk of toxicity and minimizing the selective pressure for resistance development. This strategy is supported by the work of Hancock and Sahl, who emphasized the potential of antimicrobial peptides as antibiotic adjuvants in combating multidrug-resistant pathogens.^34^

It is worth noting that while the combinations showed improved activity against some Gram-negative bacteria, they remained less effective against Kp. This highlights the ongoing challenge of developing broad-spectrum treatments for highly resistant Gram-negative pathogens, as discussed by Theuretzbacher et al. in their review of the current antibiotic pipeline.^48^

Further exploration of proline modifications led to the development of RW6-2P, a variant with two proline residues incorporated into the RW6 sequence. The results of this modification are presented in Table 8.

**Table 8:** Antimicrobial Activity and Toxicity of RW6-2P and Its Combinations with Antibiotics^a^

Peptide/combination	MRSA	*E. coli*	Kp	Pa	Ab	HEK293	RBC
RW6-2P	≤0.25	16	>32	≤0.25	2	>32	>32
VAN-rw6-2p	2	32	32	>32	32	>32	>32
PEN-rw6-2p	≤0.25	32	>32	>32	32	>32	>32
AMP-rw6-2p	4	>32	>32	>32	>32	>32	>32

a All values are MICs in μg/mL.

The incorporation of two proline residues in RW6-2P resulted in a unique activity profile. RW6-2P maintained potent activity against MRSA (MIC ≤ 0.25 μg/mL) and showed improved efficacy against Pa (MIC ≤ 0.25 μg/mL) compared to its single-proline counterpart. This enhanced activity against Pa is particularly noteworthy, as this pathogen is known for its intrinsic resistance to many antibiotics. The improved efficacy might be attributed to the increased conformational flexibility induced by the two proline residues, allowing better penetration of the robust outer membrane of Pa. This observation aligns with the findings of Scocchi et al., who reported that proline-rich antimicrobial peptides can effectively target Gram-negative bacteria by penetrating their cell membranes and inhibiting intracellular targets.^49^

However, activity against *E. coli* was reduced (MIC: 16 μg/mL) compared to RW6P, suggesting that the optimal number of proline residues may vary depending on the target pathogen. This variability in efficacy across different bacterial species is consistent with the work of Li et al., who demonstrated that the positioning and number of proline residues in antimicrobial peptides can significantly influence their spectrum of activity.^50^

Importantly, RW6-2P maintained low toxicity to human cells (MIC > 32 μg/mL for both HEK293 and RBCs), aligning with previous studies showing that proline incorporation can enhance selectivity for bacterial membranes over mammalian cells. This selectivity is crucial for developing antimicrobial peptides with improved therapeutic indices, as highlighted by Tossi et al. in their comprehensive review of proline-rich antimicrobial peptides.^51^

When combined with conventional antibiotics, RW6-2P showed synergistic effects with PEN against MRSA (MIC ≤ 0.25 μg/mL), but the combinations were less effective against Gram-negative bacteria. This synergistic effect with β-lactam antibiotics against Gram-positive bacteria is consistent with the findings of Brogden et al., who reported that proline-rich peptides can enhance the efficacy of conventional antibiotics by facilitating their entry into bacterial cells.^35^

These findings underscore the complex relationship between peptide structure and antimicrobial activity and highlight the potential of multiproline modifications in developing targeted antimicrobial peptides with reduced toxicity. The work of Knappe et al. on the optimization of proline-rich antimicrobial peptides further supports the potential of this approach in designing next-generation antimicrobial agents.^52^

In summary, the synergistic effects observed between (RW)*n* peptides, particularly RW6P and RW6-2P, and conventional antibiotics offer a promising approach to combating antimicrobial resistance. These combinations may provide a way to extend the useful life of existing antibiotics and offer new treatment options for difficult-to-treat infections.

## Conclusion

This study demonstrates the potent antimicrobial activity of (RW)*n* peptides, particularly RW6, and highlights the enhanced efficacy achieved through proline modification and peptide-antibiotic combinations. RW6 and its proline-modified variants show great promise as next-generation antimicrobials, offering strong activity against Gram-positive bacteria and, with some structural adjustments, the potential for improved efficacy against Gram-negative pathogens. The favorable toxicity profiles observed in this study further support the potential therapeutic use of these peptides. Future research should prioritize optimizing the structural design of these peptides to enhance their activity against Gram-negative bacteria, investigating their efficacy in combination with other antibiotics in clinical applications, and exploring their potential as conjugates with antibiotics to improve therapeutic outcomes. This approach could significantly contribute to overcoming the global challenge of antimicrobial resistance.

## Supporting Information Available

MIC values of the peptides and their mixtures (PDF)

## Abbreviations

Ab: *Acinetobacter baumannii*
AMPs: Antimicrobial Peptides
AMP: Ampicillin
CO-ADD: Community for Open Antimicrobial Drug Discovery
DMSO: Dimethyl sulfoxide
Ec: *Escherichia coli*
HPLC: High-Performance Liquid Chromatography
Kp: *Klebsiella pneumoniae*
MDR: Multidrug-Resistant
MRSA: Methicillin-Resistant *Staphylococcus aureus*
Pa: *Pseudomonas aeruginosa*
PDR: Pan-Drug Resistant
PEN: Penicillin
RBCs: Red Blood Cells
RWn: Arginine and tryptophan Peptide Series
VAN: Vancomycin
WAAW: World Antimicrobial Resistance Awareness Week
XDR: Extensively Drug-Resistant
