# Supplementary material for: Proline-Modified (RW)n Peptides: Enhancing the Antimicrobial Efficacy and Selectivity against Multidrug-Resistant Pathogens
Source: ACS Omega. 2025 Feb 7;10(10):10450–8. doi: 10.1021/acsomega.4c10757 (PMC11923837; doi:10.1021/acsomega.4c10757)
Supplement: Supplementary file 1 — ao4c10757_si_001.pdf [file ao4c10757_si_001.pdf]

Proline-Modified (RW)n Peptides: Enhancing Antimicrobial Efficacy and Selectivity Against Multidrug-Resistant Pathogens

Anderson Sunda-Meya[1] ** and Nsoki Phambu*[2]

[1]Department of Physics, Xavier University of Louisiana, New Orleans, LA 70125, USA

[2]Department of Chemistry, Tennessee State University, Nashville, TN 37209, USA

Supporting information

Table 1S. MIC values of the peptides (with or without proline) and their mixtures with or without antibiotics (component in capital letter means 95 mg and component in minor letter means 5 mg).

**Compound**	**Sa**	**Ec**	**Kp**	**Pa**	**Ab**	Hk	Hm
RW4	8	>32	>32	>32	>32	>32	>32
RW6	<=0.25	<=0.25	>32	>32	>32	>32	<=0.25
Rw8	2	32	>32	>32	32	>32	0.4523
**RW4P**	<=0.25	<=0.25	>32	>32	>32	>32	>32
**RW6P**	<=0.25	>32	>32	>32	>32	>32	>32
**RW8P**	<=0.25	>32	>32	>32	8	>32	>32
**RW6-2P**	<=0.25	16	**>32**	<=0.25	2	>32	>32
VAN-rw6-2p	2	32	32	>32	32	>32	>32
PEN-rw6-2p	<=0.25	32	>32	>32	32	>32	>32
AMP-rw6-2p	4	>32	>32	>32	>32	>32	>32
PEN	8	32	>32	>32	>32	>32	>32
AMP	32	8	>32	>32	>32	>32	>32
VAN	<=0.25	>32	>32	>32	>32	>32	28.69
PEN-rw4	8	>32	>32	>32	>32	>32	>32
PEN-rw6	8	32	>32	>32	>32	>32	>32
PEN-rw8	16	>32	>32	>32	>32	>32	>32
VAN-rw4	1	32	>32	>32	32	>32	>32
VAN-rw6	2	32	>32	>32	>32	>32	8.943
VAN-rw8	1	>32	>32	>32	32	>32	1.827
AMP-rw4	4	16	>32	>32	>32	>32	>32
AMP-rw6	16	16	>32	>32	>32	>32	>32
AMP-rw8	16	32	>32	>32	>32	16.75	>32
RW4-Pen	8	32	>32	32	>32	>32	3.949

RW6-Pen	4	32	>32	>32	32	>32	4.414
RW8-Pen	8	>32	>32	>32	32	>32	0.621
RW4-Van	32	32	>32	32	32	>32	2.846
RW6-Van	4	16	>32	>32	32	30.69	0.742
RW8-Van	8	>32	>32	>32	>32	20.26	0.467
RW4-Amp	32	32	>32	32	32	>32	24.49
RW6-Amp	2	16	>32	>32	32	>32	0.661
RW8-Amp	16	32	>32	>32	32	19.54	0.773
**RW6P**							
PEN-rw6p	<=0.25	>32	>32	>32	>32	>32	>32
AMP-rw6p	8	32	>32	>32	>32	>32	>32
VAN-rw6p	<=0.25	>32	>32	>32	>32	>32	>32
RW6P-pen	<=0.25	>32	>32	>32	>32	>32	>32
RW6P-amp	<=0.25	<=0.25	>32	>32	>32	>32	>32
RW6P-van	<=0.25	<=0.25	>32	>32	8	>32	>32
PEN-rw4p	2	32	>32	>32	>32	>32	>32
AMP-rw4p	2	8	>32	>32	32	>32	>32
VAN-rw4p	<=0.25	32	32	>32	16	>32	>32
PEN-rw8p							
AMP-rw8p	2	8	>32	<=0.25	>32	>32	>32
VAN-rw8p							
